# *PLOS Neglected Tropical Diseases*: Ten years of progress in neglected tropical disease control and elimination … More or less

**DOI:** 10.1371/journal.pntd.0005355

**Published:** 2017-04-20

**Authors:** Peter Hotez, Serap Aksoy

**Affiliations:** 1 Texas Children’s Hospital Center for Vaccine Development, National School of Tropical Medicine at Baylor College of Medicine, Houston, Texas, United States of America; 2 Department of Biology, Baylor University, Waco, Texas, United States of America; 3 Center for Health and Biosciences, James A. Baker III Institute for Public Policy, Rice University, Houston, Texas, United States of America; 4 Scowcroft Institute of International Affairs, Bush School of Government and Public Service, College Station, Texas, United States of America; 5 Department of Epidemiology of Microbial Diseases, Yale School of Public Health, Yale University, New Haven, Connecticut, United States of America

## Abstract

This year *PLOS Neglected Tropical Diseases* (*PLOS NTDs*) celebrates its tenth anniversary following the publication of the first issue in 2007 [1]. When *PLOS NTDs* was founded, the framework of the neglected tropical diseases (NTDs) as an alternative to “other diseases” (as they were then referred to in the Millennium Development Goals) was just getting started—especially for Africa [2, 3]. In the decade since, *PLOS NTDs* has overseen enormous successes in NTD control and elimination. Here, we want to briefly review the ten year progress made towards the control or elimination of the diseases now identified by the WHO as NTDs. Many of the details are highlighted in *PLOS NTDs* papers cited here, but the summary information is based on the recently released Global Burden of Disease (GBD) Study 2015 (also launched with Gates Foundation support) that summarized past-decade changes in disease prevalence, mortality, or disability rates (from the years 2005 to 2015) [4–6], as well as the GBD Study 2013 that summarizes disease prevalence changes over a longer time horizon from 1990 to 2013 [7].

The most common NTDs are the three major intestinal helminth infections, schistosomiasis, and scabies. Each is associated with more than 100 million of cases globally, followed by tens of millions of cases each of dengue, food-borne trematode infections, lymphatic filariasis (LF), and onchocerciasis. Between 1 million and 10 million people are affected by Chagas disease, leishmaniasis, trachoma, cysticercosis, and cystic echinococcosis, while thousands are affected by leprosy, rabies, human African trypanosomiasis (HAT), Ebola virus infection, and yellow fever. There are no specific GBD data for the other diseases that comprise the WHO list of NTDs, such as Buruli ulcer, yaws, dracunculiasis, and mycetoma. As shown in Box 1, we can group the major NTDs into diseases that are headed towards elimination, those for which we have made significant versus minimal gains, and diseases for which we are falling behind.

**Box 1**

- Heading towards elimination
  ○ Dracunculiasis
  ○ Human African trypanosomiasis (HAT)
  ○ Lymphatic filariasis (LF)
  ○ Trachoma
  ○ Yaws
- Significant gains
  ○ Rabies
  ○ Leprosy
  ○ Onchocerciasis
  ○ Ascariasis
  ○ Schistosomiasis
  ○ Cysticercosis
- Minimal gains
  ○ Trichuriasis
  ○ Hookworm disease
  ○ Food-borne trematodiases
  ○ Cystic echinococcosis
- Losing the battle
  ○ Dengue and other arbovirus infections
  ○ Leishmaniasis
  ○ Chagas disease

## Heading towards elimination

There are now five NTDs for which disease elimination is a realistic goal over the next decade, if not sooner. Global advocacy for these disease elimination efforts are currently being organized under the auspices of a London Declaration for NTDs created in 2012 [8]. They include disease elimination targets for HAT, LF, trachoma, and yaws, and the eradication of dracunculiasis. Over the last decade, there has been more than a 75% reduction in the mortality rates (and cases) of HAT, mostly for the Gambian form of the disease. These gains have been achieved through case detection and treatment and tsetse control [9]. The “last mile” of HAT elimination will require working in conflict and postconflict nations of sub-Saharan Africa, including Central African Republic, Chad, Democratic Republic of Congo, and South Sudan. Attempts to control Rhodesian form of the HAT disease will require further efforts given that the disease has extensive animal reservoirs, which serve as a source of the parasite for tsetse fly transmission.

For LF and trachoma, we have seen more than 50% decreases each in their global prevalence, mostly through WHO-led mass drug administration (MDA) and allied measures (including morbidity management for LF and the surgery for trichiasis, antibiotics, facial cleanliness, and environmental improvement [SAFE] strategy for trachoma), such that the elimination of these two diseases is also now considered a realistic target [10, 11]. However, success in global elimination for both LF and trachoma assumes that international funds for MDA will continue. It has been further observed that azithromycin used in trachoma elimination efforts could simultaneously result in progress towards yaws elimination [11].

## Significant gains

The last decade has also seen significant gains for another half dozen NTDs, which could also result in progress towards elimination. Deaths from canine rabies (responsible for more than 95% of the deaths from rabies) have decreased more than 50% over the last decade, and there is a blueprint shaped by global experts for its eventual elimination [12]. Similarly, there has been tremendous progress in global leprosy elimination efforts through multidrug therapy, although it has been pointed out that there remains a large segment of the population that still suffers from leprosy in endemic areas and remains both undiagnosed and untreated [13].

MDA has also produced enormous gains. Since 1990, the global prevalence of onchocerciasis has decreased by more than 50% through community-directed treatment with ivermectin under auspices of the African Programme for Onchocerciasis Control (APOC) [14], with parallel elimination efforts in the Americas. APOC has now reorganized to add more NTDs targeted through MDA. MDA using either donated mebendazole or albendazole (including the albendazole used as part of LF elimination efforts [15]) is beginning to reduce the global prevalence of ascariasis (approximately 20% over the last decade), while a more recent industry donation of praziquantel is beginning to show results for prevalence reductions (approximately 30% over the last decade) in schistosomiasis. However, we have also seen the emergence or reemergence of schistosomiasis in new areas, including Corsica in southern Europe. Global efforts are underway to determine how MDA for schistosomiasis can be expanded and to fold in additional measures that could accelerate control or elimination, including snail control and antischistosome vaccines among other interventions [16, 17]. Finally, praziquantel used for schistosomiasis MDA is starting to create spillover effects to reduce the prevalence of taeniasis in Africa, although it is generally believed that a more integrated “one health” approach will be required to achieve global elimination of cysticercosis [18].

## Minimal gains

In contrast to the important successes highlighted above, minimal gains have been achieved for other high prevalence NTDs. Disease prevalence reductions through MDA for ascariasis so far have not translated to similar reductions for trichuraisis or hookworm infection. For trichuriasis, the fact that single-dose albendazole or mebendazole often does not produce cures or significant intensity reductions as it does for ascariasis is likely an important factor, although combining albendazole with either oxantel pamoate or ivermectin may help overcome this deficiency [19]. For hookworm infections, single-dose albendazoe or mebendazole is also variable in its efficacy [20], and there is the additional and important problem that hookworm exhibits high prevalence and intensities among adults not currently targeted by MDA [21]. Therefore, new or improved technologies or control approaches may be required [17]. Similarly, so far there have been no coordinated and well-funded programs for the control of food-borne trematodiases or cystic echinococcosis, although a new international alliance has been created for scabies.

## Losing the battle

Finally, there is a group of NTDs that has increased significantly in prevalence or remained mostly unchanged over the last decade. Most of them are vector-borne NTDs. For instance, there has been a dramatic uptick in the incidence of dengue and other arbovirus infections, including chikungunya and Zika virus infection, as well as Ebola virus infection, a zoonotic NTD [22]. The increased incidence of one or more of these diseases has been especially noted in the Americas, southern Europe, the Middle East, and Asia, and could be due to a number of factors, including virus mutations, host population expansions and human migrations, urbanization and deforestation, and climate change [22]. Conflicts in the Middle East and East Africa may also explain the significant rise of leishmaniasis that has been noted in the Middle East and East Africa [23, 24]. For Chagas disease, there has been no significant decrease in the global prevalence since earlier *Triatoma infestans* vector control efforts produced significant declines in the Southern Cone of South America. Recently, benznidazole, the major drug used to treat Chagas disease, was shown to have no therapeutic impact on patients with chagasic cardiomyopathy. There also remains an urgent need to develop better Chagas disease technologies [25].

## Concluding statements

The last decade has seen a mixed picture when it comes to success stories in the progress to control or eliminate the world’s NTDs. There is great hope that a group of at least five NTDs could be eliminated or eradicated within a few years, with continued significant gains for at least six other NTDs. However, for roughly an equal number of diseases progress has been minimal or stalled or, in some cases, we are losing ground, especially for some of the vector-borne and zoonotic NTDs [26]. On the positive side, the last decade has seen an explosion in the genetic and genomic knowledge gained on NTD pathogens and vectors. This fundamental knowledge has fueled the development of new drug targets, vaccines, and vector control methods that will play a most important role for the control of NTDs in the next decade. There remains an urgent need to coordinate global efforts against the NTDs and to press for continued investments in both implementation and research and development. At *PLOS NTDs* we look forward to our role in the next decade on this front and thank the global NTD community of experts for their unwavering support.
